# Understanding Empathy Toward Dissimilar Others in Challenging Everyday Interactions

**DOI:** 10.1002/hbm.70283

**Published:** 2025-07-23

**Authors:** Rui Watanabe, Hironobu Kuruma

**Affiliations:** ^1^ Turku PET Centre and Turku University Hospital, University of Turku Turku Finland; ^2^ Tokyo Metropolitan University Tokyo Japan

## Abstract

Empathy is essential for human social interaction; however, extending empathy toward individuals with dissimilar characteristics facing daily challenges may be difficult. This study investigated how people without disabilities empathize with individuals with disabilities, specifically those with stroke‐induced hemiplegia, during manual interactions with objects or other people. Using functional magnetic resonance imaging and multivoxel pattern analysis (MVPA), we examined the neural and behavioral mechanisms underlying empathy in these contexts. Participants observed video stimuli featuring individuals with hemiplegia performing hand movements, such as grasping a human hand or an object (a plastic bottle), using either their hemiplegic or nonhemiplegic hands. Behavioral results showed that observing grasping movements performed by the hemiplegic hand elicited stronger negative empathic feelings than those performed by the nonhemiplegic hand, regardless of the grasping targets. Positive empathic feelings were more pronounced while observing the hemiplegic hand grasping the human hand than the object. Furthermore, classification approaches in MVPA revealed that parts of the mirror neuron system and mentalizing networks distinguished empathic responses to grasping the human hand and the object commonly across the hemiplegic and the nonhemiplegic hands conditions. Additionally, the dorsal medial prefrontal cortex (MPFC) more accurately classified empathic responses to hemiplegic than nonhemiplegic grasping movements. Representational similarity analysis revealed that brain regions associated with affective empathy were specifically attuned to feelings of relief involved in the grasping movements across conditions. These findings suggest that both affective and cognitive empathic brain systems are mutually engaged when empathizing with individuals with hemiplegia who face complex challenges. The dorsal MPFC likely plays a key role in facilitating precise empathic responses to the challenges of hemiplegic movements. Moreover, the affective system is particularly fine‐tuned to positive feelings, such as relief. Our findings advance understanding of the neural mechanisms underlying empathy toward individuals with different characteristics.

## Graphical abstract

We revealed that empathy toward individuals with physical disabilities engages distinct neural mechanisms involving both affective and cognitive empathy, as shown by fMRI and MVPA. In particular, the dorsal MPFC plays a key role in processing empathy for individuals facing challenges, while the anterior insula is linked to positive feelings elicited by observed behavior.

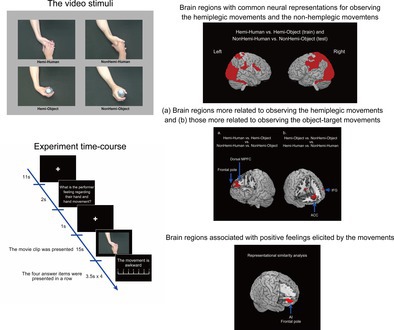

## 1 Introduction

Human empathy is a fundamental aspect of the social experience in scientific literature, yet people are occasionally less empathic toward others (Decety and Porges 2011). (Gamble et al. 2024) Reportedly, people struggle to empathize with those who have characteristics different from their own, such as dissimilar physical appearances, races, and life experiences (Aziz‐Zadeh et al. 2012; Forgiarini et al. 2011; Herrera et al. 2018). Hence, empathizing with a target person without sharing representations stemming from similar physical or mental experiences is difficult. Additionally, a distant relationship with the target individual or lack of knowledge about them may reduce empathic response toward them (Gamble et al. 2024; de Waal and Preston 2017; Zaki and Ochsner 2012). In fact, our previous studies highlighted that lay people without disabilities have greater difficulty empathizing with individuals who have physical impairments than physiotherapists who work daily with these individuals in rehabilitation. The dissimilar physical representations and unfamiliarity of lay individuals with those having disabilities reduce their empathic response toward them (Watanabe et al. 2019).

However, in another study, even though lay people without disabilities find it difficult to fully empathize with individuals with disabilities facing challenging situations, they are still capable of leveraging the brain system underlying empathy, which allows them to empathize to some extent (Watanabe et al. 2022). In our study, we demonstrated that people without disabilities show specific brain activity patterns in cognitive and affective empathic regions when observing simple hand movements performed using the paralyzed hands. The affective system is engaged intuitively in sharing another person's emotional experience and motor representation, whereas the cognitive system is responsible for reflectively mentalizing another person's thoughts (de Waal and Preston 2017; Bernhardt and Singer 2012; Shamay‐Tsoory 2011). Potentially, these systems may foster a deeper sense of empathy toward individuals with dissimilar characteristics when they work together and mutually enhance each other. Notably, the challenges faced by individuals with disabilities are often complex and unfamiliar to those without disabilities; thus, relying on both empathic systems is essential to feel their emotional and mental states (Watanabe et al. 2022).

In our previous study, we focused on intransitive hand movements: clasping‐unclasping movements. However, in daily life, we typically encounter and observe transitive actions or meaningful behaviors involving hands, such as manipulating objects or interacting with other individuals. These behaviors are possibly more closely associated with everyday life and engage more complex cognitive processes (Buccino et al. 2001; Cheng et al. 2006). Importantly, several brain networks are activated to process the superficial information and underlying contexts when observing a person performing specific actions, such as interacting with an object or another person (Brass et al. 2007; Spunt et al. 2011; Iacoboni et al. 2004).

These transitive behaviors for individuals with physical disabilities are more complex in both physical and mental aspects. In particular, individuals with stroke‐induced hemiplegia face significant challenges when performing such interactions using their paralyzed hand. Tasks involving the paralyzed hand in daily activities are notably difficult (Alt Murphy and Häger 2015; Raghavan 2015; Levin et al. 2019). These difficulties are evident in the inefficient kinematics of their hand movements and their substantial need to improve hand function, compared with other stroke‐related impairments (Kowalczewski et al. 2007; Pollock et al. 2014).

We hypothesized that when people without disabilities observe those with disabilities manually interacting with objects or other people, they would engage in more complex and affective processes to empathize with those with disabilities facing the challenges. These mental processes are possibly reflected in multiple brain systems activation, involved in empathy. Therefore, in this study, we aimed to investigate how people without disabilities empathize with individuals with hemiplegia induced by stroke during interactions with objects or people, using functional magnetic resonance imaging (fMRI); accordingly, we sought to clarify the neural mechanisms that underlie empathy toward individuals with differing characteristics in daily social situations.

Thoroughly identifying the underlying neural mechanisms using multivoxel pattern analysis (MVPA) is crucial to determining the process of empathic response toward difficulties associated with manual interaction by individuals with disabilities (Haxby et al. 2001; Kriegeskorte et al. 2008). MVPA is widely utilized to identify brain activity patterns across multiple voxels, providing detailed information associated with a specific cognitive function. This method reveals information that might be undetectable using traditional univariate approaches, which focus on the overall amplitude of brain activity. Concurrently, representational similarity analysis (RSA) provides insight into neural patterns associated with behavioral variables by testing correlations across experimental conditions. Given the strengths of MVPA, we anticipated that this approach would more precisely capture the neural representation of empathic response.

## 2 Materials and Methods

Thirty‐three right‐handed participants (20 women and 13 men; mean age 20.9 ± 1.7) were included in this experiment. All participants had no history of neurological or psychiatric disorders and were verified as right‐handed using the Edinburgh Handedness Inventory (Oldfield 1971). The participants, who were nonmedical undergraduate students, nonmedical graduate students, and general office workers, were selected, excluding medical professionals or students. After the experiment, none of the participants reported any prior experience directly interacting with individuals with disabilities, including stroke patients, such as working or living with individuals with physical disabilities. In this experiment, each participant received compensation (6000 JPY).

Regarding the sample size, before starting our study, we first referred to our previous study (Watanabe et al. 2022) in which a sample size of 26 was calculated and deemed sufficient. Since the current study adopts a similar design (an fMRI study with MVPA and psychological data analysis), we considered it reasonable to follow a similar sample size. To further support this rationale, Nee (2019) showed that studies with over 40 min of total scan time across multiple sessions can achieve reliable results with approximately 23 participants. In our case, the total scan time was approximately 60 min, consisting of eight runs, further supporting the adequacy of our sample size.

From the perspective of behavioral data, we conducted a power analysis using G*power (Faul et al. 2007). For this analysis, we used the effect size from our key comparisons: Hemi‐Human versus Hemi‐Object and Hemi versus Non conditions. The smallest effect size observed was *r* = 0.42 (from the Hemi‐Object and Hemi‐Human on the item AWKWARD), yielding a statistical power of 1 − *β* = 0.99 with our sample size of 33 (*α* = 0.025).

All participants provided written informed consent, and the study protocol was approved by the Institutional Ethics Committee of our institution. The study was conducted following the Declaration of Helsinki.

### 2.1 Stimuli

For our experimental stimuli, we recorded and edited the movie clips that featured the individuals with hemiplegia: eight individuals with hemiplegia (five men and three women: mean age 58.1 ± 8.8 years). The individuals included in this movie stimuli had a moderate degree of hemiplegia and were unable to fully flex and extend their fingers: they were also unable to independently move each finger. All the individuals were classified as individuals with chronic stroke because the mean period from the onset of stroke was 5.4 ± 3.0 years. Four had left‐sided hemiplegia, and the other four had right‐sided hemiplegia.

**FIGURE 1:**
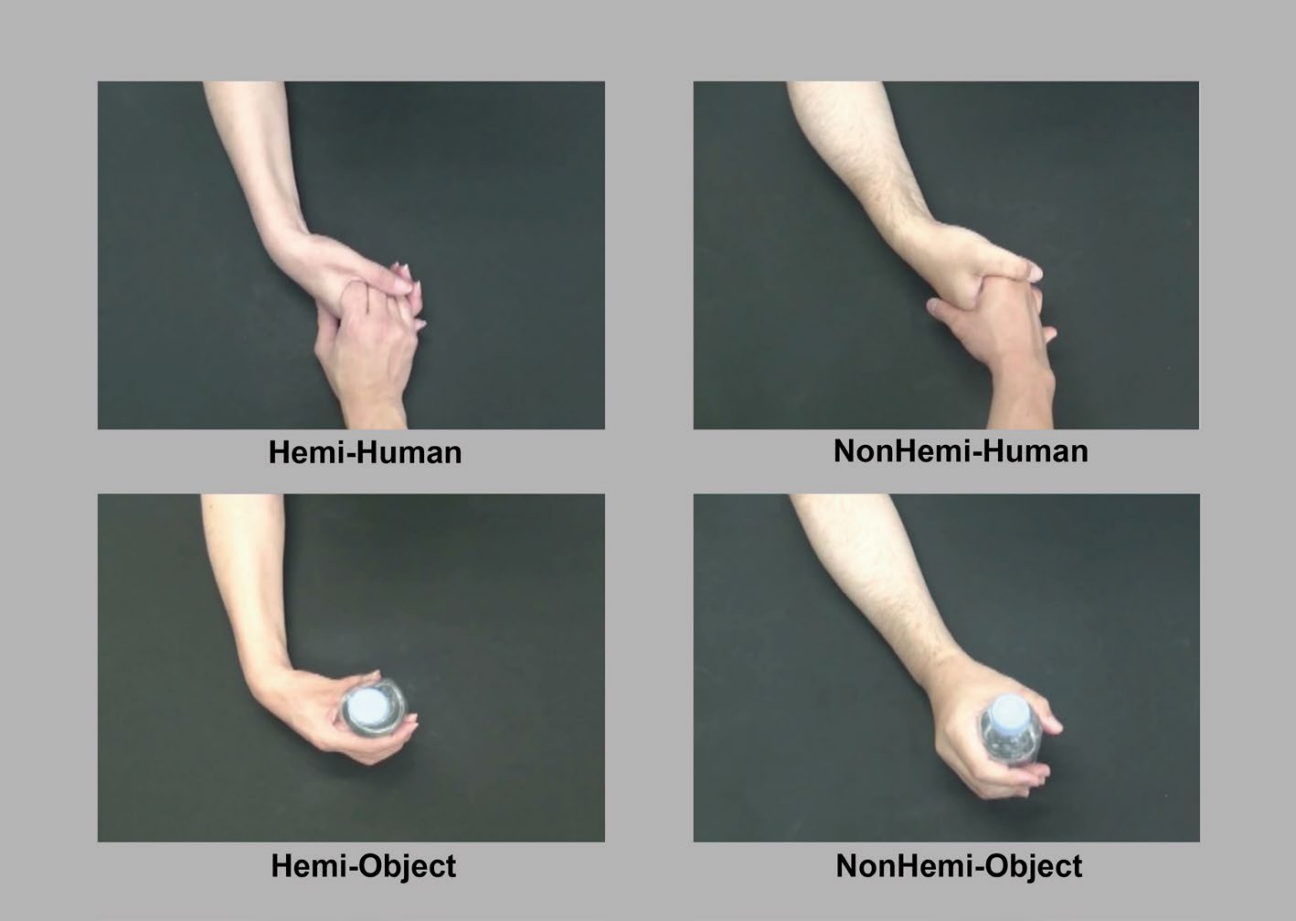
Four conditions of movie clips featuring manual interactions. In each image, the hand shown at the top belongs to the individual being empathized with. The left column shows the hand movements performed by the hemiplegic side, whereas the right column shows the hand movements performed by the nonhemiplegic side. The top row illustrates grasping a human hand, and the bottom row illustrates grasping a plastic bottle. Hemi: hemiplegic hand movements; Human: a human hand; NonHemi: nonhemiplegic hand movements; Object: an object (a plastic bottle).

In the clips, each of them grasped either a human hand from a person without disabilities (Human) or grasped a plastic water bottle (Object) using their hand on the hemiplegic side (Hemi) or the nonhemiplegic side hand (NonHemi), resulting in Hemi‐Human, Hemi‐Object, NonHemi‐Human, and NonHemi‐Object conditions (Figure 1). Generally, patients with hemiplegia attempt to use their paralyzed hands in their daily life; nevertheless, they often struggle due to the severity of their paralysis, making it difficult to control their hand as they wish. Before recording, we asked the individuals with hemiplegia about the challenging situations they encounter in their daily life. Based on their responses, we identified that they frequently have difficulty performing the two hand movements described above and then opted for them for our movie stimuli. All video clips were recorded using a digital video camera (GZ‐RX500‐B; JVC Inc., Kanagawa, Japan).

All the movie clips were recorded as if they were viewed from a third‐person perspective (as if the hand was coming from front of the participant). This was because observing the hands from this perspective could lead participants to perceive the presented hand belonging to another person (Bortoletto et al. 2013; Vistoli et al. 2016; Watanabe et al. 2017). Additionally, we aimed to investigate the neural and behavioral reactions when they observed another person with characteristics different from their own.

**FIGURE 2:**
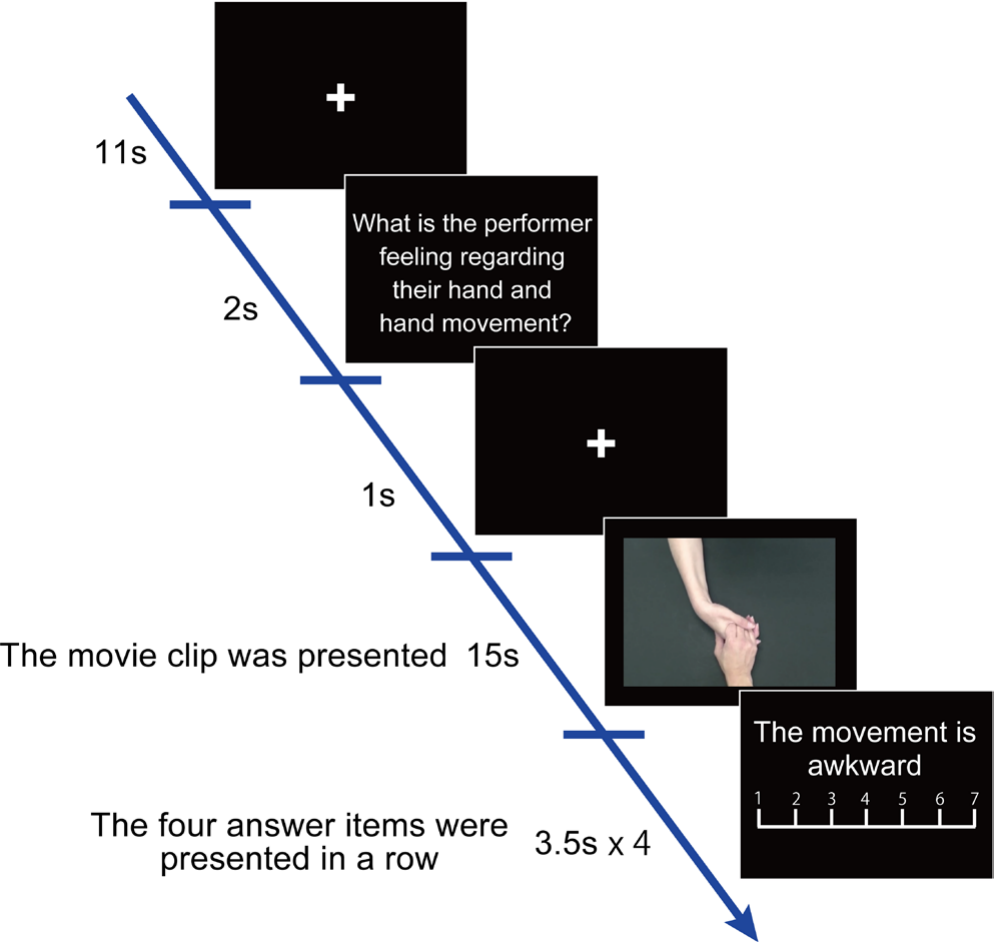
Experiment time‐course with the following sequence. The instruction, movie clips, and four answer items used in the experiment are shown.

After recording the movie clips, we adjusted each clip to a duration of 7 s by trimming the start and end phases, ensuring they included the scenes of incompletely grasping and releasing the hand or plastic bottle (Figure 2). Next, all movie clips showing left‐hand movements were flipped horizontally to appear as if the movement were performed with the right hand. The direction of the body could influence the process of assuming the perspective of another person; thus, we ensured consistency by showing all the movements as right‐hand movements. The results showed that all the movie clips comprised right‐hand movement. In total, we prepared 32 movie clips from eight individuals with hemiplegia.

Regarding low‐level visual features of our video stimuli, such as brightness or contrast, we did not extract or calculate quantitative measures. However, all videos were recorded under consistent conditions, including the same lighting, a closed windowless room, and identical video camera settings, to reduce potential differences in these features. In addition, to minimize substantial differences in motion energy across conditions, we standardized the initial hand position: in all videos, the hand was already lightly touching the target object when the video began.

### 2.2 Measurement of the Empathic Response Toward Movie Clips

During the fMRI scan, we showed movie clips featuring individuals with hemiplegia and asked the question, “What is the performer feeling regarding their hand and hand movements?” for each trial (Watanabe et al. 2022). We chose this question to intentionally prompt our participants to take the perspective of the performers to control the strategy they used. Subsequently, we presented four answer items and their descriptions, using a 7‐point scale from 1 (do not agree at all) to 7 (completely agree). The answer items comprised two negative and positive items, each accompanied by the related descriptions. The two negatives were: (1) the movement is awkward (AWKWARD); (2) the performer feels irritated by their hands and hand movements (IRRITATED). The two positives were (3) the performer feels relieved while grabbing the target (RELIEVED); (4) the performer feels they are firmly grabbing the target (FIRM).

Furthermore, we considered it rational to ask the individuals with hemiplegia who performed in our movie stimuli what they were feeling during the hand movements, as their descriptions would reflect their original feelings. The emotional experience of stroke patients is not always as clear‐cut as terms like “pain,” which is commonly used in empathy research. Therefore, we asked each of them what they were feeling while grasping the target with their hemiplegic hand. They described various feelings; however, they all reported experiencing the negative feelings of AWKWARD and IRRITATED (as described above). We also asked if they experienced any positive feelings, and all of them reported feeling RELIEVED while grasping a human hand, whereas FIRM was the predominant feeling when grasping the plastic bottle. Based on these responses, we selected these four items as our empathic answer items. When the participants responded to each negative answer item on the 7‐point scale, higher ratings (“completely agree” = score of 7) were interpreted as greater empathy toward the difficulty involved in the hemiplegic movements. For the Hemi‐Human condition (grasping a human hand with a hemiplegic hand), a high score for RELIEVED was considered a more accurate empathic response. In contrast, for the Hemi‐Object condition (grasping a plastic bottle with a hemiplegic hand), a high score for FIRM indicated a better empathic response than a low score.

Our approach measuring the empathic response was based on our previous study (Watanabe et al. 2022), in which participants watched videos of stroke patients' hand movements and reported their subjective empathic responses similar to the current study. However, in the preliminary behavioral experiment in that prior study, we found that while some participants naturally adopted the perspective of the performers (stroke patients), others did not although we had simply asked them to rate what they felt. We hypothesized that this variability may have stemmed from the unfamiliarity of the movements, which made it challenging to relate to the experience. To address this, in both the previous and current studies, we asked participants, “What is the performer feeling regarding their hand and hand movement?” This question was intended to encourage cognitive perspective‐taking and elicit empathic responses. This approach draws support not only from our earlier work but also prior empathy research (Decety et al. 2013; Borja Jimenez et al. 2020; Moriguchi et al. 2007), and is considered to encourage participants to engage in cognitive empathy.

### 2.3 Procedure

The fMRI experimental session comprised eight runs, with each run including eight trials comprising four experimental conditions presented twice. Each trial began with the presentation of a white fixation cross for 11 s (Figure 2). Subsequently, the question “What is the performer feeling regarding their hand and hand movement during their movements?” was displayed for 2 s. Immediately after this question, a 1‐s white fixation cross appeared, followed by a 7‐s movie clip from one of four conditions, which was presented twice with a 1‐s white fixation cross between them. Subsequently, the four answer items with their descriptions in response to the question, with the 7‐point scale, were sequentially presented for 3.5 s for each item. Furthermore, the order of presentation of the four items was fully randomized for each trial. During the presentation, participants were asked to verbally respond using the 7‐point scale. Next, the verbal responses were recorded via a voice‐capture microphone system (FOMRITM II, Optoacoustics Ltd., Or Yehuda, Israel) attached to the head coil for the MRI experiment.

Each run contained two Hemi‐Human trials, Hemi‐Object trials, NonHemi‐Human trials, and NonHemi‐Object trials. In total, 64 trials (16 trials for each condition) were administered to each participant. In each run, the presentation order of the trials was fully randomized, and the total fMRI session lasted approximately 60 min. Participants were not informed about who was performing the hand movements until after they completed the experimental tasks to prevent them from recognizing that the performers in the movie clips were individuals with hemiplegia. After the experiment, we confirmed that none of the participants recognized that the hand movements in the hemiplegic condition were performed by the paralyzed hands of individuals with stroke.

### 2.4 Imaging Data Acquisition

The movie clips were presented using Presentation 14.0 (Neurobehavioral Systems Inc., Albany, CA, USA) and viewed through goggles attached to the fMRI system (resolution = 800 × 600 pixels; virtual image distance, 3 m; Resonance Technology Inc., Northridge, CA, USA). The MRI data were acquired using a GE SIGNA Premier 3.0 T scanner (GE Healthcare, Waukesha, WI, USA) equipped with a birdcage‐type quadrature detection head coil and an actively shielded gradient coil. Moreover, functional scans were obtained using gradient echo and echoplanar imaging (EPI) sequences. In addition, T2*‐weighted images were acquired. The blood oxygenation level‐dependent sensitive single‐shot EPI sequence parameters were as follows: repetition time (TR) = 1500 ms; echo time (TE) = 16.4 ms; flip angle (FA) = 70°; matrix size = 64 × 64 pixels; field of view (FOV) = 200 × 200 mm; slice number = 65; slice thickness = 2.0 mm; and slice gap = 0.3 mm. For each participant, we acquired a T1‐weighted anatomical image with the following parameters: TR = 6.7 ms; TE = 3 ms; FA = 8°; matrix size = 256 × 256; FOV = 240 × 240 mm; and slice thickness = 1 mm.

### 2.5 Data Analyses for Subjective Empathic Ratings

We used MATLAB 2021a (Mathworks Inc., Sherborn, MA) for the analysis of the subjective empathic ratings. The main dependent variables were the subjective empathic rating scores for each answer item, which were verbally measured as the participants underwent the fMRI scan. Furthermore, we conducted a Wilcoxon signed rank test to compare the median differences of each answer item across the four experimental conditions: Hemi‐Human versus Hemi‐Object, Hemi‐Human versus NonHemi‐Human, Hemi‐Object versus NonHemi‐Object, and NonHemi‐Human versus NonHemi‐Object. For each test, the significance level was set at α < 0.0125. For this threshold, Bonferroni correction was performed to avoid type I error (i.e., 0.05 was divided by four, the number of above comparisons).

### 2.6 Functional Magnetic Resonance Imaging Data Analyses

#### 2.6.1 Preprocessing

Image data processing was performed using SPM12 software (Wellcome Department of Cognitive Neurology, http://www.fil.ion.ucl.ac.uk/spm) implemented in MATLAB. For each participant, the EPI images were initially realigned to the first image to adjust for motion‐related artifacts. Subsequently, slice timing correction was used to align each image to the middle slice. The mean of the realigned EPI images was further co‐registered with the T1‐weighted magnetic resonance (MR) images. The co‐registered T1‐weighted images were subsequently normalized to the Montreal Neurological Institute (MNI) template. The EPI images were not normalized and smoothed to avoid blurring the fine‐grade information contained in the multivoxel activity before implementing MVPA (Hebart et al. 2014; Weaverdyck et al. 2020). We used the general linear model (GLM) to estimate beta coefficients for our four experimental conditions (the 16‐s period during which the movie clips were presented). Each condition was modeled using a boxcar function and convolved with the canonical hemodynamic response function. Additionally, the models included realignment parameters as confounding variables, and the run‐wise estimated beta coefficients for each condition were leveraged to the subsequent MVPA.

For univariate analysis, after coregistration, the parameters of the normalization process for T1‐weighted images were applied to each EPI image. The processed EPI images were spatially smoothed using a Gaussian kernel of 8 mm full‐width at half‐maximum.

#### 2.6.2 Multivoxel Pattern Analyses: Classification Analyses

We conducted classification analyses on the EPI data to identify multivoxel patterns using the decoding toolbox (TDT) (Hebart et al. 2014). For this analysis, a whole‐brain searchlight approach was applied, using 8 mm‐radius spheres centered around each voxel for all voxels. Furthermore, each participant's brain was partitioned into overlapping voxel clusters using the created spheres. In each of these clusters, we subsequently employed a linear support vector machine (SVM) using LIBSVM implementation (http://www.csie.ntu.edu.tw/~cjlin/libsvm/) with default settings, including a fixed regularization parameter of C = 1, to calculate decoding accuracies. Consistent with these procedures, we used cross‐validation and cross‐classification approaches for each participant. The cross‐validation approach was employed to identify brain regions that encode (1) the effect of different targets (a human hand versus an object) involved in the difficult manual interactions and (2) the effect of hemiplegic movements (hemiplegic hand vs. nonhemiplegic hand) during these interactions, when empathizing with individuals with disabilities facing challenges associated with such interactions. To this end, binary classifiers were trained to capture the neural empathic responses that vary depending on the differences in the target of interaction.

These classifiers were based on two comparisons: (1) Hemi‐Human versus Hemi‐Object and NonHemi‐Human versus NonHemi‐Object, and (2) Hemi‐Human versus NonHemi‐Human and Hemi‐Object versus NonHemi‐Object. For each comparison, the SVM was trained on seven out of eight runs and tested on the remaining untrained runs, yielding the average classification accuracy. This process was iteratively performed until every run had been tested (an eight‐fold leave‐one‐run‐out cross‐validation approach). These processes generated four accuracy maps per participant, with each voxel reflecting the average proportion of correctly classified runs. On the other hand, the cross‐classification approach was employed to identify brain areas that reflect the common neural representation when empathizing with interactions involving both hemiplegic and nonhemiplegic hands. We trained the classifier on the Hemi‐Human versus Hemi‐Object contrast and evaluated its performance on the NonHemi‐Human versus NonHemi‐Object contrast. The value of each voxel in each map represents the average proportion of correctly classified runs.

Each of the mean accuracy maps resulting from the two approaches was spatially normalized to the standard MNI brain template using the parameters obtained through co‐registration and subsequently smoothed using the 8 mm full width at half maxima Gaussian kernel. For the second‐level analysis for the cross‐validation approach, the smoothed images were entered into a random effect analysis using a 1 × 2 factorial design, where the factor ‘hemiplegia’ was modeled as a within‐subject factor. We compared the decoding accuracies of (Hemi‐Human vs. Hemi‐Object) with those of (NonHemi‐Human vs. NonHemi‐Object). Additionally, we examined the difference in the decoding accuracies between Hemi‐Human versus NonHemi‐Human and those of Hemi‐Object versus NonHemi‐Object using a factorial design, with the factor “targets” modeled as a within‐subject factor. For the cross‐classification, we used a random‐effect analysis to investigate the voxels with significantly higher accuracy than the chance level (50%).

#### 2.6.3 Multivoxel Pattern Analyses: RSA

RSA identifies the brain regions where the neural activity patterns across pairs of the four experimental conditions significantly correlate with the behavioral rating patterns for the corresponding pairs of each answer item. This analysis can determine brain regions that reflect the negative or positive feelings represented in subjective empathic score (Kriegeskorte et al. 2008; Popal et al. 2019). To this end, we used brain imaging data from the periods during which movie clips were presented in the Hemi‐Human and Hemi‐Object conditions separately. First, we created a 4 × 4 behavioral representational dissimilarity matrix (RDM) for each answer item based on the average scores from the four experimental conditions for each participant (Figure S1). Furthermore, the value in each cell of the behavioral RDM was calculated using the Euclidean distance between each pair of the experimental conditions based on the average scores for each answer item.

We subsequently created a 4 × 4 neural RDM and compared it with the behavioral RDM to identify the brain regions whose representation structure resembles that of the behavioral RDM, using the whole‐brain searchlight approach for each Hemi condition separately. An 8 mm‐radius sphere was created around each voxel, followed by extracting beta estimates for each of the four experimental conditions. Subsequently, we calculated the correlation coefficient *r* between the vectors of beta estimates for each pairwise comparison of the four experimental conditions. The dissimilarity value between each pair of conditions was subsequently calculated as 1 minus the *r*‐value. This process resulted in creating a 4 × 4 neural RDM representing the dissimilarities between all conditions. Each participant's neural RDM was compared with each behavioral RDM using Spearman correlation. The resulting correlation coefficient was then converted to a z‐value using Fisher's transformation to meet statistical assumptions. We ultimately created continuous statistical whole‐brain maps that reflect how well the subjective empathic responses, based on the focus answer item toward hemiplegic movements, are represented in the neural activity patterns. For each answer item, we examined the relationship between behavioral data and brain activity separately for each Hemi condition. The resulting whole‐brain maps were spatially normalized and smoothed in the same manner as the classification analyses after conducting RSA for each participant.

For the second‐level analyses, each participant's whole‐brain map, obtained from the first‐level analyses in each classification approach and RSA, was entered into second‐level analyses for each approach. The statistical threshold was set at *p* < 0.001 (uncorrected) at the voxel‐wise level and *p* < 0.05 (corrected for family‐wise error) at the cluster‐wise level. All coordinates were reported in MNI space. The anatomical locations of significant clusters were labeled based on the SPM Anatomical Toolbox (Eickhoff et al. 2005) and the Automated Anatomical Labeling atlas (Tzourio‐Mazoyer et al. 2002).

#### 2.6.4 Univariate Analysis

The first‐level participant‐wise analysis was conducted using the GLM with the hemodynamic response function modelled as a boxcar function for the four conditions of interest. The included conditions of interest were Hemi‐Human, Hemi‐Object, NonHemi‐Human, and NonHemi‐Object. The realignment parameters were also included in the GLM as confounding variables. For each calculation, each participant's first‐level contrasts were introduced into the second‐level random‐effects analysis using a 2 × 2 factorial design, where the factor “hemiplegia” and “targets” were modeled as a within‐subject factor. We compared condition‐specific parameter estimates as follows: the main effect of hemiplegia (1) Hemi‐Human + Hemi‐Object versus NonHemi‐Human + NonHemi‐Object and (2) NonHemi‐Human + NonHemi‐Object versus Hemi‐Human + Hemi‐Object, the main effect of targets (3) Hemi‐Human + NonHemi‐Human versus Hemi‐Object + NonHemi‐Object and (4) Hemi‐Object + NonHemi‐Object versus Hemi‐Human + NonHemi‐Human, and the interaction effect (5) (Hemi‐Human vs. Hemi‐Object) versus (NonHemi‐Human vs. NonHemi‐Object) and (6) (Hemi‐Object vs. Hemi‐Human) versus (NonHemi‐Object vs. NonHemi‐Human). The statistical threshold for these analyses was set at *p* < 0.001 (uncorrected) at the voxel level and *p* < 0.05 (corrected for family‐wise error) at the cluster level.

## 3 Results

### 3.1 Scores of the Answer Items

**FIGURE 3:**
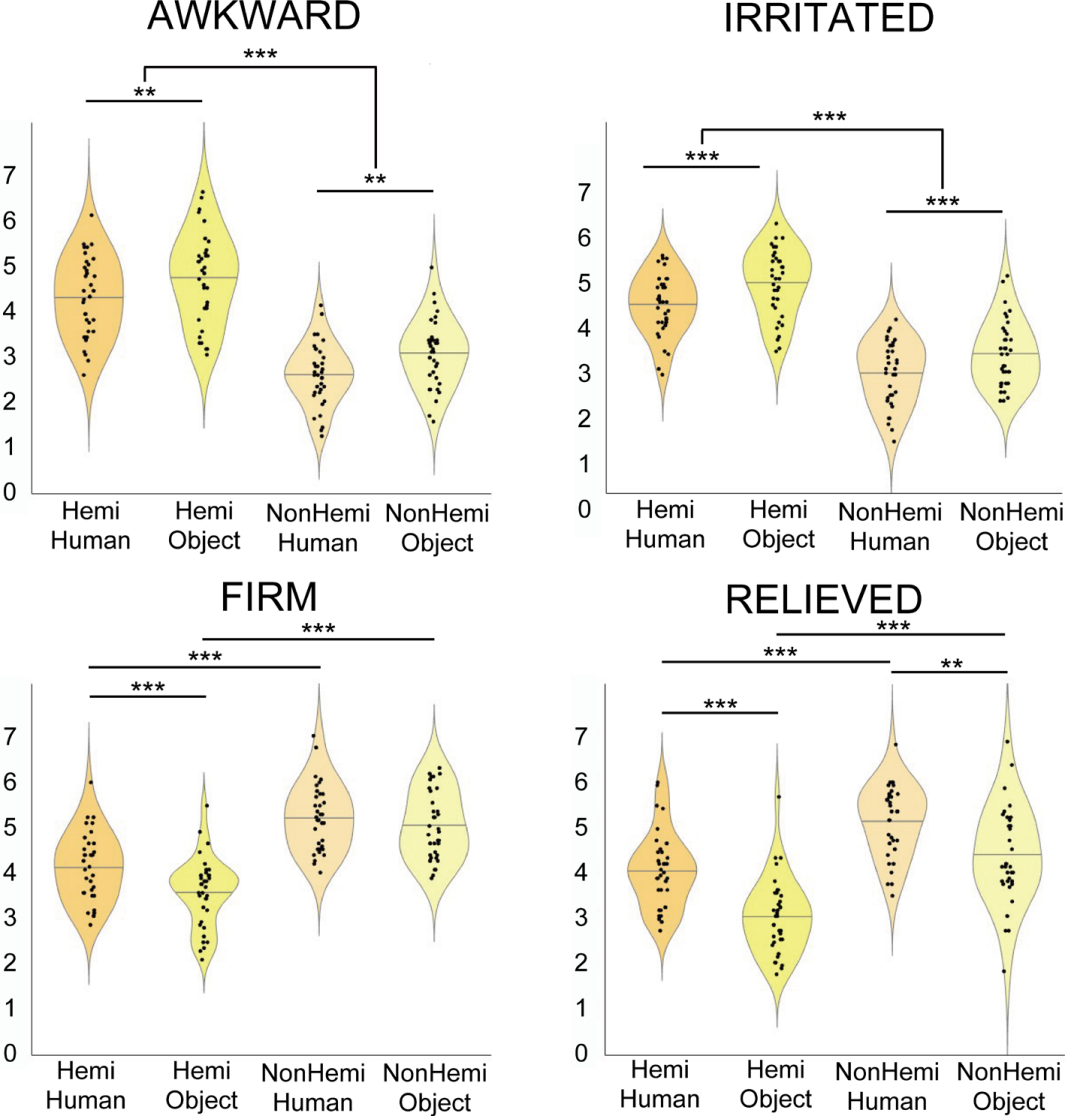
Violin plots illustrating the subjective empathic scores for the four answered items across the four experimental conditions. Each violin represents the data distribution for each condition, with individual data points overlaid and a horizontal line indicating the median score. Significant differences between conditions are indicated by asterisks, with *p* < 0.01 represented using ** and *p* < 0.001 represented using ***. Hemi: hemiplegic hand movements; Human: a human hand; NonHemi: nonhemiplegic hand movements; Object: an object.

Each subjective empathic median score for the four answer items (two negative items, AWKWARD and IRRITATED, and two positive items: FIRM and RELIEVED) among the four conditions is shown in Figure 3.

We performed a Wilcoxon signed‐rank test with FDR correction to compare the median differences of each answer item across the four experimental conditions: Hemi‐Human versus Hemi‐Object, Hemi‐Human versus NonHemi‐Human, Hemi‐Object versus NonHemi‐Object, and NonHemi‐Human versus NonHemi‐Object. In the negative items, significant differences were found in AWKWARD and IRRITATED across multiple conditions. In AWKWARD, ratings were significantly higher in Hemi‐Object than Hemi‐Human (*p* < 0.005, *r* = 0.42), Hemi‐Human than NonHemi‐Human (*p* < 0.001, *r* = 0.74), and Hemi‐Object than NonHemi‐Object (*p* < 0.001, *r* = 0.86). The difference between NonHemi‐Human and NonHemi‐Object did not reach significance (*p* = 0.03, *r* = 0.38). In IRRITATED, all condition pairs showed significant differences: Hemi‐Object was rated higher than Hemi‐Human (*p* < 0.001, *r* = 0.51), Hemi‐Human was higher than NonHemi‐Human (*p* < 0.001, *r* = 0.86), Hemi‐Object was higher than NonHemi‐Object (*p* < 0.001, *r* = 0.81), and NonHemi‐Object was higher than NonHemi‐Human (*p* < 0.01, *r* = 0.42).

In the positive items, significant differences were observed in FIRM and RELIEVED. In FIRM, ratings were significantly higher for Hemi‐Human than Hemi‐Object (*p* < 0.001, *r* = 0.65) and for NonHemi‐Object than Hemi‐Object (*p* < 0.001, *r* = 0.87). Additionally, NonHemi‐Human was rated higher than Hemi‐Human (*p* < 0.001, *r* = 0.78). However, the comparison between NonHemi‐Human and NonHemi‐Object was not significant (*p* = 0.51, *r* = 0.16). In RELIEVED, all condition pairs showed significant differences: Hemi‐Human was rated higher than Hemi‐Object (*p* < 0.001, *r* = 0.77), NonHemi‐Object was higher than Hemi‐Object (*p* < 0.001, *r* = 0.85), NonHemi‐Human was higher than Hemi‐Human (*p* < 0.001, *r* = 0.76), and NonHemi‐Human was higher than NonHemi‐Object (*p* < 0.01, *r* = 0.48). Full statistical results are provided in Table S1.

### 3.2 Searchlight Classification Analyses

**FIGURE 4:**
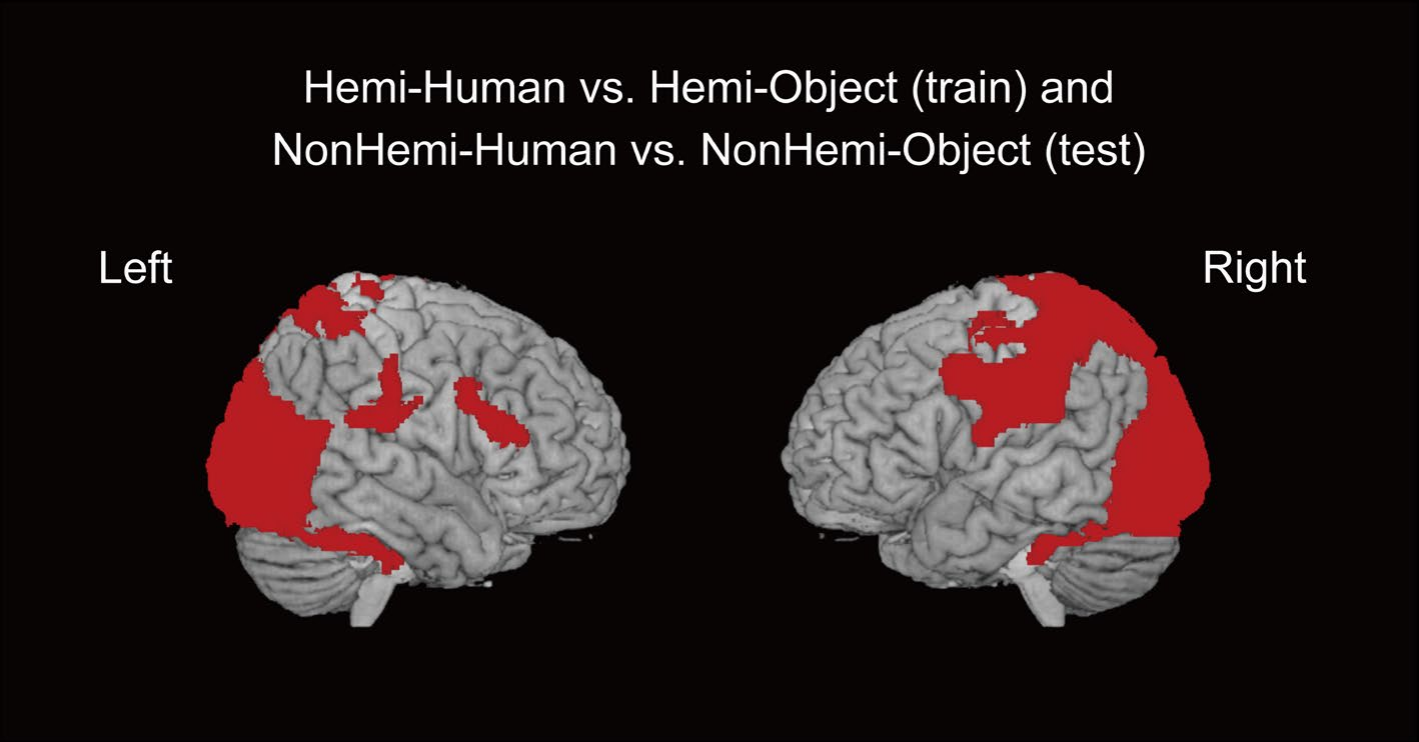
Brain regions identified through the cross‐classification approach. The images show neural activity patterns that reflect the common neural representation between Hemi‐Human versus Hemi‐Object and NonHemi‐Human versus NonHemi‐Object. The classifiers were trained on Hemi‐Human versus Hemi‐Object and then tested on NonHemi‐Human versus NonHemi‐Object. Hemi: hemiplegic hand movements; Human: a human hand; NonHemi: nonhemiplegic hand movements; Object: an object.

We addressed searchlight MVPA to detect brain activity patterns involved in empathizing with individuals with hemiplegia interacting with the human hand and with the object using cross‐validation and cross‐classification approaches. For the cross‐classification approach, the classifiers were trained on Hemi‐Human versus Hemi‐Object and subsequently evaluated on NonHemi‐Human versus NonHemi‐Object. The second‐level analysis further revealed significantly distinct activity patterns in the large cluster from the left and right front‐parietal areas to the temporal and visual areas (Figure 4 and Table 1). These findings suggest that these brain areas differentiate empathic responses when empathizing with the manual interactions involving the human hand and the object, commonly when performed by the hemiplegic side hand and nonhemiplegic side hand.

**TABLE 1:** Brain regions identified through the cross‐classification approach, showing neural activity patterns that reflect the common neural representation between Hemi‐Human versus Hemi‐Object and NonHemi‐Human versus NonHemi‐Object.

L/R	Region	MNI coordinates				
		*x*	*y*	*z*	*t* value	Voxels
R	Inferior frontal gyrus	40	28	12	4.72	781
L	Premotor area	−58	4	38	5.75	63,085
L	Sensory motor area	−56	−20	36	5.58	
R		30	−34	60	3.89	
L	Inferior parietal lobule	−54	−26	36	7.96	
R		26	−52	54	5.23	
L	Superior parietal lobule	−10	−78	44	10.9	
R		22	−54	60	6.19	
R	Temporoparietal junction	50	−58	18	4.27	
L	Superior temporal gyrus	−54	−30	22	4.58	
R		54	−32	22	4.18	
L	Precuneus	−8	−80	44	11.09	
R		10	−72	32	6.44	
L	Fusiform gyrus	−34	−68	−10	13.66	
R		32	−66	−14	10.18	
L	Lingual gyrus	−10	−86	−2	16.67	
R		18	−82	0	12.48	
L	Associate visual areas	−4	−86	10	20.6	
R		18	−84	10	22.06	

*Note:* The threshold was set at *p* < 0.001 (uncorrected) for the voxel level and *p* < 0.05 (family‐wise error, corrected) for the cluster level. The minimum cluster size was 781.

Abbreviations: Hemi: hemiplegic hand movements; Human: a human hand; L: left; NonHemi: nonhemiplegic hand movements; Object: an object; R: right.

**FIGURE 5:**
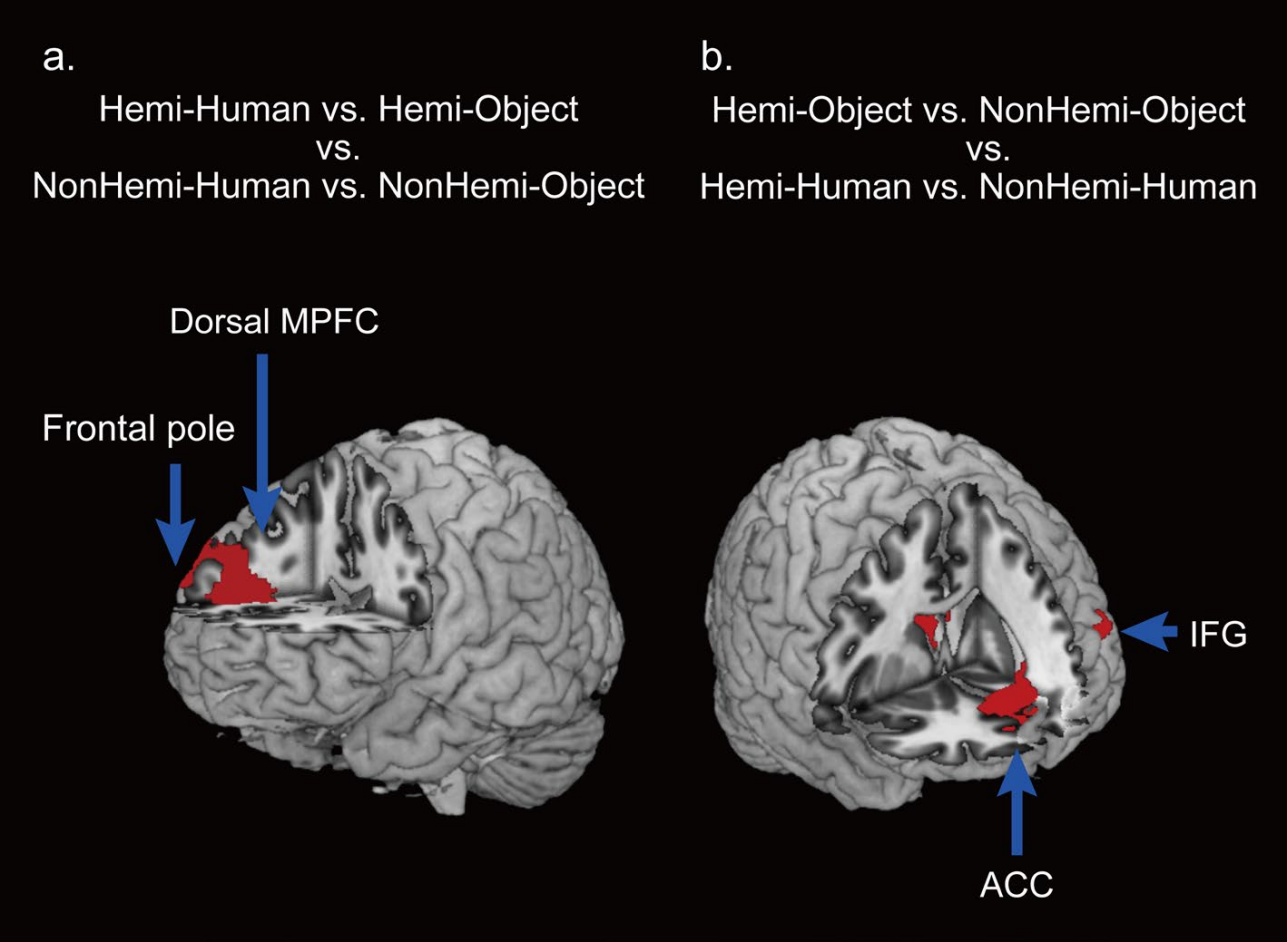
Brain regions identified using the cross‐validation approach. (a) Brain regions that more accurately classified Hemi‐Human versus Hemi‐Object compared with NonHemi‐Human versus NonHemi‐Object. (b) Brain regions that more accurately classified Hemi‐Object versus NonHemi‐Object compared with Hemi‐Human versus NonHemi‐Human. ACC: anterior cingulate cortex; Hemi: hemiplegic hand movements; Human: a human hand; IFG: inferior frontal gyrus; MPFC: medial prefrontal cortex; NonHemi: nonhemiplegic hand movements; Object: an object.

For the cross‐validation approach and its second‐level analysis ([Hemi‐Human vs. Hemi‐Object] versus [NonHemi‐Human vs. NonHemi‐Object]), we observed significantly more accurate neural activity patterns in the right frontal pole and the dorsal medial prefrontal cortex (MPFC) (Figure 5a and Table 2). These brain activity patterns classified empathic responses when empathizing with the individuals with hemiplegia interacting with the human hand and with the object, which are performed by the hemiplegic side hand more accurately than those performed by the nonhemiplegic side hand. The opposite contrast ([NonHemi‐Human vs. NonHemi‐Object] versus [Hemi‐Human vs. Hemi‐Object]) showed significantly specific activity patterns in the inferior parietal lobule (IPL) and early visual areas (Table 2). This neural activity more accurately identified empathic responses while empathizing with the manual interactions in the nonhemiplegic condition than in the hemiplegic condition.

**TABLE 2:** Brain regions identified through the cross‐validation approach (comparisons between Hemi and NonHemi conditions).

L/R	Region	MNI coordinates			*t* value	Voxels
(a) Brain regions that more accurately classified Hemi‐Human vs. Hemi‐Object compared with NonHemi‐Human vs. NonHemi‐Object.						
		*x*	*y*	*z*		
R	Frontal pole	34	42	26	4.88	1006
R	Medial prefrontal cortex	22	38	20	6.83	
(b) Brain regions that more accurately classified NonHemi‐Human vs. NonHemi‐Object compared with Hemi‐Human vs. Hemi‐Object.						
		*x*	*y*	*z*		
L	Early visual areas	−12	−84	20	4.52	1493
R	Inferior parietal lobule	70	−16	34	4.06	557

*Note:* The threshold was set at *p* < 0.001 (uncorrected) for the voxel level and *p* < 0.05 (family‐wise error, corrected) for the cluster level. The minimum cluster sizes were 1006 voxels for (Hemi‐Human vs. Hemi‐Object) versus (NonHemi‐Human vs. NonHemi‐Object) and 557 voxels for (NonHemi‐Human vs. NonHemi‐Object) versus (Hemi‐Human vs. Hemi‐Object).

Abbreviations: Hemi: hemiplegic hand movements; Human: a human hand; L: left; NonHemi: nonhemiplegic hand movements; Object: an object; R: right.

We again performed the cross‐validation approaches and their second level analyses to find the specific brain regions related to each target of manual interaction, (Hemi‐Object vs. NonHemi‐Object) versus (Hemi‐Human vs. NonHemi‐Human) and (Hemi‐Human vs. NonHemi‐Human) versus (Hemi‐Object vs. NonHemi‐Object). We detected that specific activity patterns in the left anterior cingulate cortex (ACC), left thalamus, and left inferior frontal gyrus (IFG) significantly more accurately identified the movements in the Object condition than in the Human condition (Figure 5b and Table 3). Meanwhile, we discovered that specific activity patterns only in the calcarine cortex significantly more accurately classified the hemiplegic and nonhemiplegic movements when interacting with the human hand than with the object in the opposite contrast (Table 3).

**TABLE 3:** Brain regions identified through the cross‐validation approach (comparisons between Object and Human conditions).

L/R	Region	MNI coordinates			*t* value	Voxels
(a) Brain regions that more accurately classified Hemi‐Object vs. NonHemi‐Object compared with Hemi‐Human vs. NonHemi‐Human.						
		*x*	*y*	*z*		
R	Anterior cingulate cortex	6	34	14	4.88	859
L		−12	34	−6	4.54	
L	Thalamus	−12	20	8	4.59	409
L	Inferior frontal gyrus	−40	36	14	4.43	494
(b) Brain regions that more accurately classified Hemi‐Human vs. NonHemi‐Human compared with Hemi‐Object vs. NonHemi‐Object.						
		*x*	*y*	*z*		
L	Calcarine cortex	−8	86	12	4.55	399

*Note:* The threshold was set at *p* < 0.001 (uncorrected) for the voxel level and *p* < 0.05 (family‐wise error, corrected) for the cluster level. The minimum cluster sizes were 409 voxels for (Hemi‐Object vs. NonHemi‐Object) versus (Hemi‐Human vs. NonHemi‐Human) and 399 voxels for (Hemi‐Human vs. NonHemi‐Human) versus (Hemi‐Object vs. NonHemi‐Object).

Abbreviations: Hemi: hemiplegic hand movements; Human: a human hand; L: left; NonHemi: nonhemiplegic hand movements; Object: an object; R: right.

### 3.3 Representational Similarity Analyses

**FIGURE 6:**
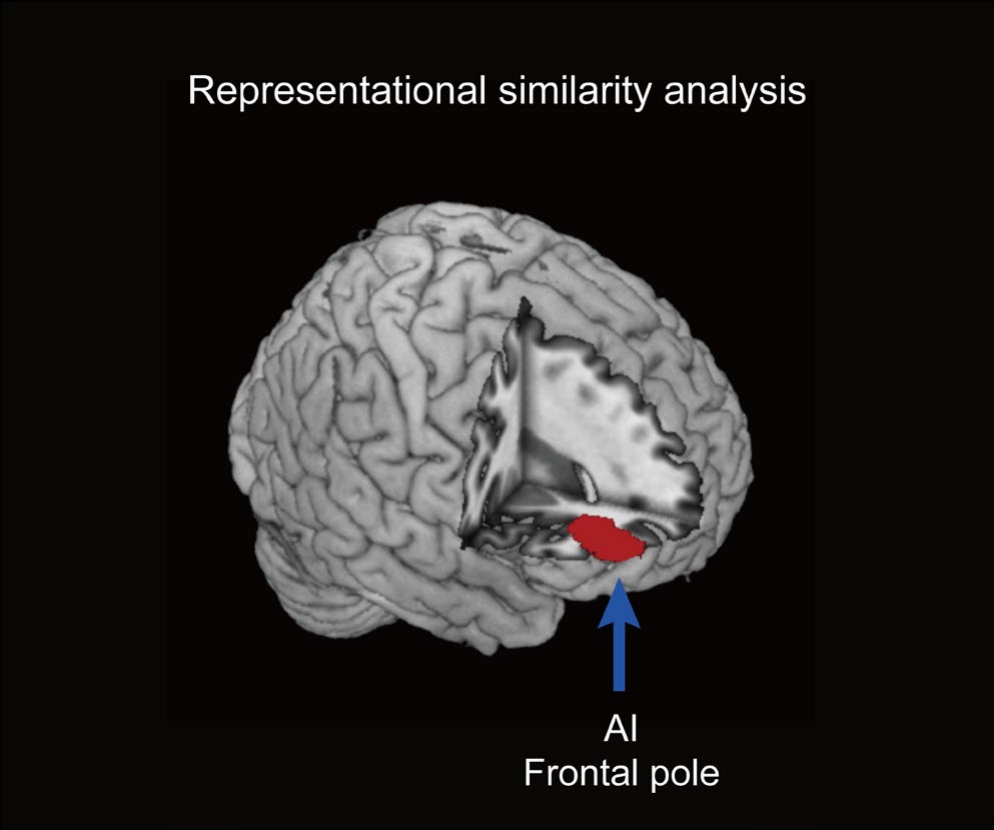
Brain regions identified using representational similarity pattern. Brian regions exhibiting significant correlations between the neural activity patterns across pairs of the four experimental conditions and that of the subjective rating patterns for RELIEVED for the corresponding pairs. These correlations indicate regions where the neural and subjective rating score patterns demonstrate strong similarity. AI: anterior insula.

For each subjective empathic item, we aimed to detect brain regions where activity patterns represent the characteristics of the target answer item connected to subjective empathy through RSA. For the item RELIEVED, we found a significant correlation in the cluster, including the anterior insula (AI), IFG, and frontal pole (Figure 6 and Table 4). However, for the other items: AWKWARD, IRRITATED, and FIRM, no correlated activation patterns were found for each. These results suggest that the subjective rating scores for RELIEVED varied across the four experimental conditions (Hemi‐Human, Hemi‐Object, NonHemi‐Human, and NonHemi‐Object), and that these changes were associated with variations in specific neural activity patterns in these brain regions across conditions.

**TABLE 4:** Brian regions exhibiting significant correlations between the neural activity patterns across pairs of the four experimental conditions and that of subjective rating patterns for RELIEVED for the corresponding pairs.

L/R	Region	MNI coordinates				
		*x*	*y*	*z*	*t* value	Voxels
R	Anterior insula	38	32	6	3.59	1412
R	Inferior frontal gyrus	50	48	6	3.56	
R	Frontal pole	42	54	0	3.47	

*Note:* The threshold was set at *p* < 0.001 (uncorrected) for the voxel level and *p* < 0.05 (family‐wise error, corrected) for the cluster level. The minimum cluster sizes were 1412 voxels.

Abbreviations: L: left; R: right.

### 3.4 Univariate Analysis

We did not find any significant activation across the main effects and interaction effects contrasts.

## 4 Discussion

In our study, we investigated how people without disabilities empathize with those with disabilities who face challenges, such as manually interacting with a human hand or an object, using fMRI and MVPA. Through this study, we sought to understand how people empathize with individuals who differ from themselves when these individuals encounter difficult situations in everyday life. Based on subjective empathic responses, the median scores for AWKWARD and IRRITATED (negative items) were significantly higher in the Hemi conditions than in the NonHemi conditions, across both grasping targets (the human hand and the object). In addition, these median scores in each negative item were significantly greater in the Hemi‐Object condition than in the Hemi‐Human condition. Meanwhile, the scores for FIRM and RELIEVED (positive items) were significantly higher in the Hemi‐Human condition than in the Hemi‐Object condition, while the scores in both Hemi conditions were lower than in both NonHemi conditions.

Notably, in the brain imaging results, neural activity patterns extending from the left and right front‐parietal regions to the temporal and visual areas significantly and accurately distinguished empathic responses when observing the manual interaction with the human hand and with the object commonly across the Hemi and NonHemi conditions. Meanwhile, neural activity patterns in the right frontal pole and the dorsal MPFC significantly more accurately identified empathic responses toward manual interactions with the human hand and with the object in the Hemi condition than those in the NonHemi condition. Particularly, activity patterns in the ACC, thalamus, and IFG significantly more precisely identified empathic responses to hemiplegic and nonhemiplegic movements in the Object condition than in the Human condition. From the RSA, activity patterns in the AI, IFG, and frontal pole were associated with the subjective empathic scores of RELIEVED, which varied across the four experimental conditions. This finding indicated that these brain regions' activity patterns were linked to the scores of RELIEVED depending on each condition.

Given that the participants scored significantly higher in each of AWKWARD and IRRITATED for the hemiplegic movements than for the nonhemiplegic movements, the participants were able to perceive the negative affective information conveyed by the hemiplegic movements to a certain degree. This finding aligns with our previous studies, which show that even lay people without disabilities empathize with, to some extent, intransitive hand movements performed by inidividuals with stroke (Watanabe et al. 2019; Watanabe et al. 2022). In the current study, using a bottle of water or a human hand as the targets of manual interaction likely made the hemiplegic movements appear more complex than simple intransitive movements. Whether this complexity made our empathic task easier or more challenging compared with that using simple intransitive movements is still unclear; however, these findings suggest that people without disabilities are capable of perceiving the difficulty involved in everyday movements of individuals with disabilities.

Meanwhile, considering the higher score in RELIEVED in the Hemi‐Human condition compared to the Hemi‐Object condition, the interaction between the hemiplegic hand and another human hand likely evoked a subjective positive empathic response associated with feeling relieved. Notably, the hemiplegic performers in the video stimuli reported feeling relieved only during interactions between their hemiplegic hand and another's hand, suggesting that the participants more precisely empathized with this affective aspect. Supporting this finding, the RSA highlighted that across each experimental condition, the scores of RELIEVED were tuned to the activity patterns in the AI and IFG, regions that significantly contribute to affective empathy. The AI is involved in interoceptive awareness of another person's emotions, and the IFG is also connected to recognizing the other's emotional states (Bernhardt and Singer 2012; Jabbi et al. 2007; Sterzer and Kleinschmidt 2010). Consistent with previous studies indicating that a handshake provides a sense of security to those involved and to observers (Levav and Argo 2010; Dolcos et al. 2012). This effect may be linked to the affective empathic brain system, resulting in a sense of relief toward the interaction between the hemiplegic hand and another's hand.

Meanwhile, the scores for FIRM in the Hemi‐Human condition were also higher than in the Hemi‐Object condition; however, the hemiplegic performers in the videos reported this sensation only when grasping an object. Considering that the performers did not experience this feeling while grasping the human hand, and given the absence of the activity patterns in brain regions linked to empathic processing, this response does not appear to reflect a genuine empathic reaction. Furthermore, this effect could have stemmed from the positive impact of the handshake itself.

Based on the brain imaging result, when the participants felt an individual interacting with either the human hand or the object, the bilateral front‐parietal‐occipital network, including the mirror neuron system (MNS) and the right temporoparietal junction (TPJ) played a pivotal role in the empathic response commonly across the Hemi and NonHemi conditions. The MNS is associated with affective empathy: this system is engaged in sharing a target person's physical state through the observer's body representation, enabling the observer to intuitively perceive what the target is feeling (Shamay‐Tsoory 2011; Carr et al. 2003; Rizzolatti et al. 2009). Meanwhile, the right TPJ is associated with cognitive empathy: it works to infer another person's mental state (de Waal and Preston 2017; Davare et al. 2006; Spunt et al. 2011). These findings reveal that regardless of whether a target person has disabilities or not, when trying to empathize with them interacting with a human hand or an object, both affective and cognitive empathy systems guide the empathic response. This is consistent with the previous studies that highlight that when observing another person doing some behavior with some objects and inferring their background information, co‐activation in the MNS and mentalizing network is exhibited (Brass et al. 2007; Spunt et al. 2011). Importantly, the fronto‐parietal activity observed here also overlaps with the action observation network, including the MNS, which is known to respond to the simple observation of others' movements (Caspers et al. 2010). Thus, part of the detected activity may reflect the perceptual processing of grasping behavior itself. However, given that action observation serves as a fundamental basis for empathy (de Waal and Preston 2017; Keysers et al. 2010), this overlap also supports the interpretation that multiple brain systems are jointly engaged in empathic processing toward individuals with disabilities. Consequently, several brain systems associated with empathy should work mutually when feeling into and inferring the transitive actions performed by either hemiplegic or nonhemiplegic hands.

Meanwhile, when empathizing with the manual interaction performed by hemiplegic hands, activity in the right dorsal MPFC and the frontal pole is more prominently involved in the empathic response than the interactions performed by nonhemiplegic hands. The MPFC plays a central role in mentalizing alongside the right TPJ. It is engaged in both self and other judgment, particularly with the dorsal region more strongly implicated in the reflective evaluation of others' emotional states (Denny et al. 2012; Olsson et al. 2007; Olsson and Ochsner 2008). Moreover, the frontal pole is similarly involved in distinguishing between self and others, aiding in the appraisal of another's mental state by integrating information about another person's internal body state with their higher‐level mental states (Olsson and Ochsner 2008; Hallam et al. 2014). Considering these findings, inferring the mental states behind manual interaction performed by hemiplegic hands likely requires more reflective inference and evaluation. For individuals without disabilities, the appearance and movements of individuals with stroke were unfamiliar, making this empathic process more demanding. Therefore, in addition to the coordinated activity between the MNS and mentalizing network, activity in the dorsal MPFC and frontal pole likely complements this network, contributing to more precise subjective empathic responses.

Additionally, specific activity patterns in the ACC, IFG, and thalamus were more strongly associated with empathic response toward interactions with the object than with the human hand ([Hemi‐Object vs. NonHemi‐Object] vs. [Hemi‐Human vs. NonHemi‐Human]). These regions are representative of brain areas involved in affective empathy. The ACC is engaged in perceiving others' unpleasant emotions, whereas the IFG is involved in observing another person in distressing situations and recognizing their emotions (Olsson et al. 2007; Schulte‐Ruther et al. 2007; Shamay‐Tsoory et al. 2009). Considering that our behavioral data revealed that the Hemi‐Object condition elicited more negative and less positive empathic responses than the Hemi‐Human condition, the relatively more negative feelings associated with the Hemi‐Object condition were likely reflected in the activity patterns in these brain regions. This effect could be more emphasized by comparing with the characteristics of the Human conditions that convey positive impressions associated with relief, such as a sense of security (Levav and Argo 2010; Dolcos et al. 2012). Therefore, interactions between a hemiplegic hand and an object are likely perceived as comparatively negative, represented in distinct activity patterns with affective empathy‐related brain regions.

As we aimed to minimize the differences in low‐level visual and motor information across the video stimuli, we did not observe any significant activity patterns in color‐sensitive or motion‐related visual areas. At the same time, we detected distinct activity patterns in brain regions engaged in empathy, such as the MPFC, ACC, and IFG—particularly in the Hemi or Object conditions. Therefore, even if subtle, the condition‐specific visual differences remained; they did not appear to drive the empathic response toward individuals with disabilities.

In the univariate analysis, we did not detect any significant activation differences across conditions, while MVPA uncovered condition‐specific patterns in several brain regions associated with empathy. This may indicate that participants engaged in a similar level of empathic processing across conditions, as the task consistently required them to empathize with the performer in every scenario. In addition, the difference in saliency between our conditions may have been more subtle compared to traditional empathy studies that contrast clearly distinct pain conditions and nonpain conditions such as observing a finger being cut by a knife versus not being touched at all (Cheng et al. 2007; Jackson et al. 2005). Since perceived pain intensity is a key factor in eliciting strong activation in brain regions associated with empathy, the negative feelings involved in our stimuli may have elicited weaker responses than those used in studies involving acute pain (Lamm et al. 2011). Nevertheless, the MVPA results suggest that these brain systems associated with empathy encoded condition‐specific features. While the strength of activation did not significantly differ across conditions, these regions appear to contribute to differentiating the specific characteristics of empathic processing.

Overall, our findings suggest that when people without disabilities attempt to empathize with individuals with disabilities interacting with either a human hand or an object, the coordinated activity among the brain regions within the MNS and the mentalizing network facilitates a more precise empathic response. The MNS, as part of the affective empathy systems, likely functions as a foundational system for perceiving feelings associated with the target behavior, whereas the mentalizing system, particularly the right TPJ, within the cognitive empathy system, contributes to empathizing with the mental states and other unobservable aspects behind the behavior. When the behavior is more complex or unfamiliar—essentially, when empathizing with another person is more challenging—the dorsal MPFC and frontal pole support the empathic response by prompting a more reflective process. Meanwhile, affective information—such as a less‐than‐secure impression arising from interaction with an object—is processed in key brain regions underlying affective empathy, specifically the ACC and IFG. Even when people intentionally adopt the perspective of individuals with disabilities, both cognitive and affective empathic systems work in tandem to support the empathic response.

There are some limitations to our study. We focused on empathy toward the challenges that individuals with disabilities face in their daily lives; however, our movie stimuli were simplified compared with real‐life situations. While we believed we effectively captured the empathic responses involved in part of social empathic situations, real daily life encompasses more complex information, which likely demands greater empathic effort. Hence, future studies should use more realistic stimuli as empathy targets, such as whole‐body movements in authentic social contexts. More realistic stimuli could reveal more nuanced responses that better distinguish the characteristics of each situation.

Furthermore, it is challenging to determine which factor involved in hemiplegic grasping movements plays a more critical role in eliciting an empathic response—such as the impaired motor information stemming from hemiplegic hand movements or the perceived emotional feeling. Given that our aim was to investigate how people without disabilities empathize with individuals with disabilities, we considered it essential to capture their combined effect, as this more accurately reflects the real‐life characteristics of individuals with stroke. However, to better understand the specific features that elicit empathic responses—whether it is the impairment associated with hemiplegia or task‐related effort—it will be necessary in future research to isolate these components.

Finally, we did not assess individual trait empathy using questionnaires, which may have limited our ability to examine how baseline empathic tendencies modulate neural and behavioral responses. However, our task‐specific design successfully elicited consistent neural and behavioral responses, suggesting that the observed effects were robust across participants. Nevertheless, future studies should include such measures to better account for individual differences in empathic processing.

## 5 Conclusion

We explored the underlying mechanism of empathy toward individuals with disabilities facing daily challenges from the perspective of people without disabilities. Our findings suggest that people can empathize considerably with others who have characteristics different from their own, especially when those individuals encounter more intricate situations. It may initially seem challenging to resonate with the complex difficulties faced by unfamiliar others; however, through jointly engaging multiple empathic systems, we may approach an understanding of their feelings, even if we cannot fully empathize with them.

## Author Contributions

**Rui Watanabe:** conceptualization, methodology, software, formal analysis, investigation, data curation, writing – original draft, writing – review and editing, project administration, funding acquisition. **Hironobu Kuruma:** investigation, data curation.

## Ethics Statement

This study protocol was approved by the Institutional Ethics Committee of Tokyo Metropolitan University (Approval Number 19025). The study was conducted following the Declaration of Helsinki.

## Conflicts of Interest

The authors declare no conflicts of interest.

## Supporting information


**Figure S1.** Example representational dissimilarity matrices (RDMs) used in the whole‐brain searchlight RSA. The top panel shows one neural RDM derived from voxel‐wise activity patterns using searchlight analysis. The bottom panels show four behavioral RDMs corresponding to participant's subjective rating scores: AWKWARD, IRRITATED, RELIEVED, and FIRM. These matrices are presented for illustrative purposes only; the actual analyses involved a larger set of RDMs across all participants and conditions.

**Table S1.** Results of Wilcoxon signed‐rank tests comparing subjective ratings across conditions for each item (AWKWARD, IRRITATED, FIRM, and RELIEF). The table shows median ± interquartile range (IQR) values for each condition, test statistics (W), *p* values, and effect sizes (*r*).

## Data Availability

The data that support the findings of this study are available on request from the corresponding author. The data are not publicly available due to privacy or ethical restrictions.
