# Supplementary material for: Understanding Empathy Toward Dissimilar Others in Challenging Everyday Interactions
Source: Hum Brain Mapp. 2025 Jul 23;46(11):e70283. doi: 10.1002/hbm.70283 (PMC12284904; doi:10.1002/hbm.70283)
Supplement: Supplementary file 1 — Figure S1. Example representational dissimilarity matrices (RDMs) used in the whole‐brain searchlight RSA. The top panel shows one neural RDM derived from voxel‐wise activity patterns using searchlight analysis. The bottom panels show four behavioral RDMs corresponding to participant's subjective rating scores: AWKWARD, IRRITATED, RELIEVED, and FIRM. These matrices are presented for illustrative purposes only; the actual analyses involved a larger set of RDMs across all participants and conditions. [file HBM-46-e70283-s001.docx]

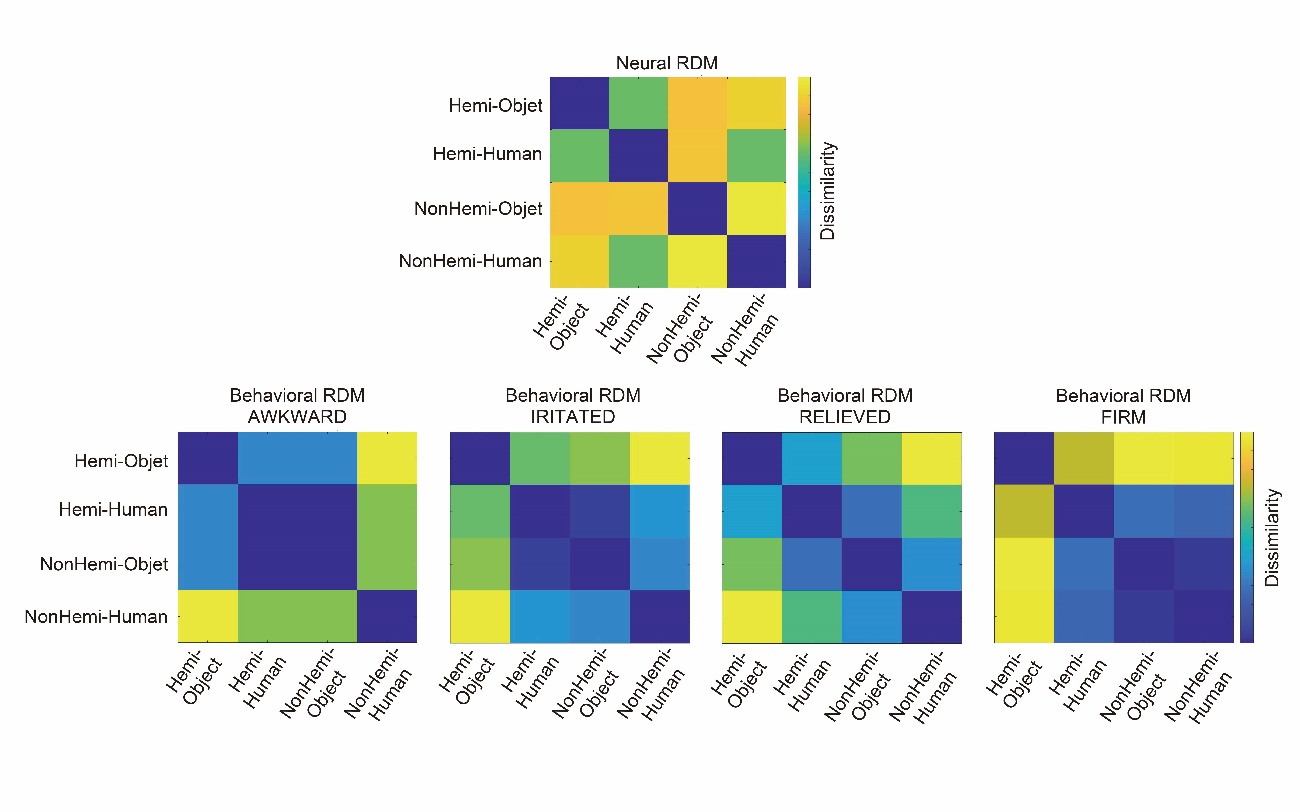
**Supplementary Figure 1**
Example representational dissimilarity matrices (RDMs) used in the whole-brain searchlight RSA. The top panel shows one neural RDM derived from voxel-wise activity patterns using searchlight analysis. The bottom panels show four behavioral RDMs corresponding to participant’s subjective rating scores: AWKWARD, IRRITATED, RELIEVED, and FIRM. These matrices are presented for illustrative purposes only; the actual analyses involved a larger set of RDMs across all participants and conditions.
