# Supplementary material for: Understanding Empathy Toward Dissimilar Others in Challenging Everyday Interactions
Source: Hum Brain Mapp. 2025 Jul 23;46(11):e70283. doi: 10.1002/hbm.70283 (PMC12284904; doi:10.1002/hbm.70283)
Supplement: Supplementary file 2 — Table S1. Results of Wilcoxon signed‐rank tests comparing subjective ratings across conditions for each item (AWKWARD, IRRITATED, FIRM, and RELIEF). The table shows median ± interquartile range (IQR) values for each condition, test statistics (W), p values, and effect sizes (r). [file HBM-46-e70283-s002.docx]

| **Item** |  | **Comparison**  **(Condition1 vs. Condition2)** | **Median ± IQR  (Condition 1)** | **Median ± IQR  (Condition 2)** | ***W*** | ***p*** | ***r*** |
| --- | --- | --- | --- | --- | --- | --- | --- |
| AWKWARD |  | Hemi-Object vs. Hemi-Human | 4.75 ± 1.13 | 4.19 ± 1.34 | 415.50 | < .005 | 0.42 |
|  |  | Hemi-Object vs. Non-Object | 4.75 ± 1.13 | 3.06 ± 0.81 | 556.50 | < .001 | 0.86 |
|  |  | Hemi-Human vs. Non-Human | 4.19 ± 1.34 | 2.63 ± 0.88 | 517.00 | < .001 | 0.74 |
|  |  | Non-Object vs. Non-Human | 3.06 ± 0.81 | 2.63 ± 0.88 | 404.00 | .03 | 0.38 |
|  |  |  |  |  |  |  |  |
| IRRITATED |  | Hemi-Object vs. Hemi-Human | 4.63 ± 1.00 | 4.13 ± 0.81 | 444.00 | < .001 | 0.51 |
|  |  | Hemi-Object vs. Non-Object | 4.63 ± 1.00 | 2.75 ± 1.06 | 540.00 | < .001 | 0.81 |
|  |  | Hemi-Human vs. Non-Human | 4.13 ± 0.81 | 2.69 ± 1.06 | 558.00 | < .001 | 0.86 |
|  |  | Non-Object vs. Non-Human | 2.75 ± 1.06 | 2.69 ± 1.06 | 417.00 | < . 005 | 0.42 |
|  |  |  |  |  |  |  |  |
| FIRM |  | Hemi-Object vs. Hemi-Human | 3.69 ± 0.75 | 4.06 ± 1.13 | 71.00 | < .001 | 0.65 |
|  |  | Hemi-Object vs. Non-Object | 3.69 ± 0.75 | 4.88 ± 1.38 | 0.00 | < .001 | 0.87 |
|  |  | Hemi-Human vs. Non-Human | 4.06 ± 1.13 | 5.06 ± 1.13 | 28.50 | < .001 | 0.78 |
|  |  | Non-Object vs. Non-Human | 4.88 ± 1.38 | 5.06 ± 1.13 | 228.50 | .51 | 0.16 |
|  |  |  |  |  |  |  |  |
| RELIEF |  | Hemi-Object vs. Hemi-Human | 3.06 ± 1 | 3.88 ± 0.83 | 33.50 | < .001 | 0.77 |
|  |  | Hemi-Object vs. Non-Object | 3.06 ± 1 | 4.13 ± 1.38 | 6.00 | < .001 | 0.85 |
|  |  | Hemi-Human vs. Non-Human | 3.88 ± 0.83 | 5.06 ± 1.13 | 37.00 | < .001 | 0.76 |
|  |  | Non-Object vs. Non-Human | 4.13 ± 1.38 | 5.06 ± 1.13 | 126.50 | < .01 | 0.48 |
|  |  |  |  |  |  |  |  |

**Supplementary Table S1**
